# Evaluation of Hemodynamic and Blood Oxygen Metabolism Alterations in Parkinson's Disease Using Quantitative MRI

**DOI:** 10.2174/011570159X385539250706094824

**Published:** 2025-12-02

**Authors:** Xinyi Lv, Rong Ye, Jie Wen, Qiang Xie, Yuhua Chen, Panpan Hu, Kai Wang

**Affiliations:** 1 Department of Neurology, The First Affiliated Hospital of Anhui Medical University, The School of Mental Health and Psychological Sciences, Anhui Medical University, Hefei, 230032, China;; 2 Department of Neurology, The First Affiliated Hospital of USTC, Division of Life Sciences and Medicine, University of Science and Technology of China, Hefei, Anhui, 230001, China;; 3 Department of Radiology, The First Affiliated Hospital of USTC, Division of Life Sciences and Medicine, University of Science and Technology of China, Hefei, Anhui, 230001, China;; 4 Department of Nuclear Medicine, The First Affiliated Hospital of USTC, Division of Life Sciences and Medicine, University of Science and Technology of China, Hefei, Anhui, 230001, China

**Keywords:** Parkinson's disease, substantia nigra, striatum, quantitative MRI, oxygen metabolism, deoxygenated cerebral blood volume

## Abstract

**Objective:**

To investigate hemodynamic and blood oxygen metabolism and their associations with disease progression, dopaminergic transporter (DAT) activity, and glucose uptake in patients with Parkinson’s disease (PD).

**Methods:**

This cross-sectional study included 73 patients with PD (mean age: 61.10 years) and 67 healthy controls (mean age: 58.99 years). Oxygen metabolism parameters—deoxygenated hemoglobin (C_deoxy_), oxygen extraction fraction (OEF_rel_), deoxygenated cerebral blood volume (dCBV), and R2* were measured using qMRI. DAT availability and glucose metabolism were assessed using PET with [18F]FP-CIT and [18F]FDG, respectively. Regional analyses were conducted using standardized brain atlases.

**Results:**

Compared with the controls patients with PD exhibited elevated C_deoxy_, OEF_rel,_ and R2* in the substantia nigra, whereas C_deoxy_ and dCBV levels were reduced in the bilateral caudate nucleus and frontal cortex (*p* < 0.05). The Hoehn-Yahr (H-Y) 2.5–3 subgroup exhibited higher levels of C_deoxy_ and OEF_rel_ in the left putamen than the H-Y 1-2 subgroup (*p* < 0.05). In the H-Y 1-2 subgroup, C_deoxy_, OEF_rel_, and R2* correlated with UPDRS scores in the substantia nigra and red nucleus (*p* < 0.05). In advanced stages (H-Y stages 2.5-3), significant correlations were observed in the striatal structures/the left dorsolateral putamen/posterior right caudate (*p* < 0.05). OEF_rel_ and R2* values were positively correlated with glucose metabolism in the left putamen and right caudate. (*p* < 0.05).

**Conclusion:**

qMRI demonstrated alterations in hemodynamics and oxygen metabolism in patients with PD, particularly within the nigrostriatal system, suggesting that metabolic indicators could serve as supplementary biomarkers for diagnosing and monitoring the progression of PD.

## 1 INTRODUCTION

Parkinson's disease (PD), the second most prevalent neurodegenerative disorder after Alzheimer's disease (AD), is a common affliction that predominantly affects middle-aged and older individuals with a considerable incidence rate [1-3]. Current diagnostic practices for PD predominantly rely on clinical symptom assessment. Still, a major lack of validated biomarkers for both diagnostic confirmation and longitudinal disease monitoring drives high rates of clinical misdiagnosis and underdiagnosis [3, 4]. The pathogenesis of PD is a complex and multifactorial process. It is characterized by the progressive degeneration of dopaminergic neurons in the substantia nigra, resulting in a reduction in dopamine levels and a consequent impairment of motor regulation [5]. Lewy bodies, abnormal protein aggregates primarily composed of α-synuclein (α-syn), are frequently detected in the brains of PD patients [6-8]. These Lewy bodies are likely to contribute to neuronal dysfunction and ultimately result in cell death [9, 10]. Furthermore, tissue hypoxia and mitochondrial dysfunction synergistically drive disease pathogenesis through oxidative stress, impaired energy metabolism (reduced ATP synthesis) [11, 12], pathological oligomerization [10, 13], and microglia-mediated neuroinflammatory cascades [14, 15], ultimately leading to nigrostriatal dopaminergic neuron degeneration and progression [16, 17]. Previous studies have also confirmed that impairments in oxygen uptake and utilization in specific brain regions may be associated with the pathogenesis and progression of PD [18].

Physiological metabolic processes include oxidative phosphorylation and aerobic glycolysis [19]. Conventional imaging techniques, such as positron emission tomography (PET) using 18F-fluorodeoxyglucose ([18F]FDG) [20] and dopamine transporter (DAT) PET [21], can, to some extent, reflect the degeneration of dopaminergic neurons and anomalies in glucose metabolism [22-24]. However, research on the imaging assessment of tissue oxygen metabolism in critical brain regions, such as the substantia nigra and striatum, in patients with PD remains limited. Traditional magnetic resonance imaging (MRI) sequences, including arterial spin labeling (ASL) [25], provide insights into local cerebral blood flow but fail to deliver information regarding tissue metabolic activity, particularly in terms of intra- and extracellular oxygen exchange. In previous studies, researchers have utilized the Gradient Echo (GRE) sequence of MRI and employed an improved multi-echo-gradient-recalled-echo (MEGRE) method to quantitatively assess tissue hemodynamic parameters, including the relative oxygen extraction fraction (OEF_rel_), deoxygenated cerebral blood volume (dCBV), and tissue concentration of deoxyhemoglobin (C_deoxy_) [26-29]. This was accomplished by differentiating the contributions of tissue (R2*) and extravascular blood oxygen level-dependent (bold) signals to the overall decay of tissue-specific GRE MRI signals (R2*). This approach has been validated for a range of neurological disorders. It has been applied to brain tissue autopsies and studies involving normal aging populations [26], multiple sclerosis [30], glioma [31], and AD [32].

This study aimed to investigate the tissue hemodynamic parameters in key brain regions implicated in PD, including the caudate nucleus, putamen, substantia nigra, red nucleus, frontal lobe, occipital lobe, temporal lobe, and other important cortical areas, using quantitative MRI techniques. The objective of this study was to investigate and evaluate the alterations in tissue oxygen metabolism and cellular architecture that affect the core motor regions in PD. Additionally, a comprehensive combined analysis of multimodal PET imaging data and clinical information was conducted to identify potential MRI biomarkers with both diagnostic and prognostic value.

## 2 MATERIALS AND METHODS

### 2.1 Subjects and Clinical Data Collection

Patients with PD were recruited from the neurology outpatient and inpatient departments of the First Affiliated Hospital of the University of Science and Technology of China between January 2021 and December 2024. The inclusion criteria were as follows: (1) Patients who met the UK Brain Bank criteria for PD [33] (Hoehn-Yahr stages 1-3). For unmedicated individuals, a direct evaluation was conducted using the Unified Parkinson's Disease Rating Scale Part III (UPDRS III) scores and a modified H-Y stage. Patients receiving medication were assessed after a 12-hour washout period. The exclusion criteria were as follows: (1) diagnosis of vascular Parkinsonism, Parkinsonism plus syndromes, drug-induced Parkinsonism, or other forms of Parkinsonism; (2) history of neurological disorders such as stroke, neuromyelitis optica, or epilepsy; and (3) unstable or severe conditions affecting the heart, lungs, liver, kidneys, or hematopoietic system. In the standard control (NC) group, age-matched healthy volunteers were included. For inclusion, participants were required to (1) be generally healthy with normal physical examination results and (2) have no history of neurological, psychiatric, or hereditary diseases and no incidents of encephalitis, tumors, brain trauma, metabolic diseases, or systemic conditions affecting the nervous system. The sample size of this study was determined in reference to the sample sizes of previous similar imaging studies [31, 32, 34], ensuring that it was sufficient to achieve the research objectives while maintaining statistical validity and reliability.

This study was approved by the Ethics Committee of the First Affiliated Hospital of the University of Science and Technology of China. It was conducted in accordance with the Declaration of Helsinki of the World Medical Association. Written informed consent was obtained from all the participants (ethical approval numbers: 2023KY-258).

### 2.2 MRI and PET Data Acquisition

A standard 24-channel head coil equipped 3-Tesla scanner (Discovery 750w; GE Medical Systems, USA) was used to conduct all MRI tests. The MRI protocol included the following sequences: (A) 3D T1 sequence with a field of view (FOV) of 256 × 256, voxel size of 1 × 1 × 1, matrix of 256 × 256, repetition time (TR) of 8.7, echo time (TE) of 3.2, and flip angle of 12. The number of excitations (NEX) was set at 1.0, the bandwidth was 31.25, and the scan time was 5 minutes and 2 s. (B) 3D multigradient echo (MGRE) sequence: FOV, 256 × 256; voxel size, 1 × 1 × 1; matrix, 256 × 256; TR, 32.1; TE, 2.1; and flip angle of 12. The bandwidth was 62.58, and the scan times were 7 minutes and 14 seconds.

The radiotracers [18F]FDG and [18F]FP-CIT were synthesized using the SIEMENS Eclipse RD cyclotron and corresponding production modules, achieving radiochemical purities over 95% (Proof of radiochemical purity in supplementary materials). Before PET/CT brain imaging, patients abstained from anti-PD medications for at least 12 h and fasted for at least 6 h. Fasting blood glucose levels were checked prior to examination, with values required to be ≤ 11.1 mmol/L. The radiotracer dosages were [18F]FDG (3.70 to 5.50 MBq/kg) and [18F] FP-CIT (3.70 MBq/kg). The subjects lay supine in a quiet environment during imaging, which was performed using a SIEMENS Biograph 16 PET/ CT scanner.

A low-dose brain CT scan was obtained for anatomical localization and PET attenuation correction, covering the area from the vertex to the lower cerebellum. The CT scan parameters were as follows: peak voltage, 120 kV; current, 300 mA; helical scanning with a slice thickness of 3.0 mm and a pitch of 0.8; an axial field of view of 30 cm; and a three-dimensional acquisition mode. Data collection began 60 minutes post-injection for [18F]FDG and 180 minutes for [18F]FP-CIT, with each acquisition lasting 10 minutes. PET images were reconstructed using TrueX, incorporating attenuation correction with a typical cross-sectional pixel size of 3 mm and a slice thickness of 3 mm. The reconstructed PET images were fused with the CT images, and all raw and reconstructed data were saved for future reference.

### 2.3 MR image and PET Data processing

#### 2.3.1 qMRI Data Processing

Quantitative analysis was performed using MATLAB 2021 (MathWorks, Natick, MA, USA) to extract tissue hemodynamic parameters, including OEF_rel_, dCBV, C_deoxy_, and R2* (Fig. **1**), based on the established qMRI method [26, 27]. To facilitate the quantitative analysis of results obtained using qMRI techniques, the Automated Anatomical Labeling (AAL) atlas and Broca's Atlas (including the regions of the red nucleus and substantia nigra) were used to delineate the regions of interest (ROIs) [35]. Additionally, finer subdivisions of the striatal structure [36, 37] (Fig. **2**). Ultimately, 20 brain regions were segmented in the Montreal Neurological Institute (MNI) stereotactic space, including deep gray matter nuclei and cortical regions. The bilateral caudate nuclei (including the anterior and posterior subdivisions), bilateral putamen (including the dorsal and ventral subdivisions), red nucleus, substantia nigra, thalamus, frontal lobe, occipital lobe, and hippocampus were extracted as ROIs to obtain the mean values of OEF_rel_, dCBV, C_deoxy_, and R2*.

#### 2.3.2 PET Data Processing

In MATLAB 2021 (MathWorks, Natick, MA), SPM5 was employed, along with the aforementioned standard-space brain template, to register and smooth the [18F]FP-CIT and [18F]FDG images with the corresponding 3D-T1 MRI images. The 3D-T1 MRI images were used for partial volume correction (PVC) of the PET images using the Müller-Gartner method [22]. Using the same brain atlas template, 20 brain regions were segmented in the MNI stereotactic space, and mean radioactivity counts of the regions of interest (ROIs) were extracted. The standardized uptake value ratio (SUVR) for [18F]FP-CIT and [18F] FDG was calculated as the ratio of the radioactivity of the ROI to that of the cerebellar gray matter.

### 2.4 Statistical Analysis

Statistical analyses were performed using IBM SPSS (version 26.0SPSS; Chicago, USA), GraphPad Prism 10.0 (GraphPad Software; La Jolla, USA), and R 4.0.4 (with the ggplot2, ggpubr, mediation, and QuantPsyc packages). The minimum sample size was determined by referencing prior multivariate neuroimaging analyses [32, 38, 39] to ensure statistical power, accounting for their higher sample size requirements. The final cohort was consistent with previous studies in effect sizes and variability. The Shapiro-Wilk test was used to evaluate normality; non-normal data were reported as median (IQR), and normal data as mean ± SD. Comparisons of continuous variables between groups were conducted using the Mann-Whitney U test for independent samples, with statistical significance defined as *p* < 0.05 for all two-sided tests. Pearson's correlation analysis was used to establish a model to explore the relationship between qMRI indicators, UPDRS scores, and multimodal PET SUVR values in the PD population, with the significance level set at *p* < 0.05.

## 3 RESULTS

### 3.1 Patient Characteristics

A total of 73 patients with PD were included in this study (mean age, 61.10 years; 56.2% female). The mean UPDRS III score was 38.48, with 34 cases classified as H-Y stage 1-2 (mean age, 60.03 years; 47.1% female) and 39 cases as H-Y stages 2.5-3 (mean age, 62.03 years; 43.2% female). The control group consisted of 67 individuals, with a mean age of 58.99 years, and 47.8% of whom were women. All the participants were right-handed. There were no statistically significant differences in sex and age between the PD, control, and PD subgroups as detailed in Table **1**.

### 3.2 Regional Assessment of Tissue Hemodynamic Parameters Extracted from qMRI in PD Core Brain Regions

Based on the ROI-based analysis, both the PD and HC groups exhibited the highest C_deoxy_ values in the bilateral pre-dorsal putamen (PD: 5.89 µM, HC: 5.59 µM). The maps of oxygen metabolism parameters (C_deoxy_, OEF_rel_, and dCBV) in the striatum revealed that oxygen metabolism in the bilateral caudate nucleus was lower than that in the bilateral putamen nucleus, demonstrating a decreasing metabolic pattern from anterior to posterior within the putamen (Supplementary Table **A1**). Compared to the HC group, the PD group exhibited elevated C_deoxy_ (3.40 µM *vs.* 2.98 µM, *p* = 0.038), OEF_rel_ (0.31 *vs.* 0.24, *p* = 0.047), and R2* values (26.67 S^-1^ *vs.* 25.4S^-1^, *p* = 0.016) in the substantia nigra region. Additionally, the C_deoxy_ and dCBV values were significantly lower in the bilateral caudate regions, particularly in the bilateral precommissural caudate and frontal cortex (*p* < 0.05), as shown in Fig. (**3**) and Supplementary Table **A1**. Within the PD group, the H-Y 2.5-3 subgroup showed increased C_deoxy_ (5.33 µM *vs.* 3.77 µM, *p* = 0.001), dCBV (0.046% *vs.* 0.036%, *p* = 0.028), OEF_rel_ (0.318 *vs.* 0.418, *p* = 0.037), and R2* values (23.63 S^-1^ *vs.* 22.31 S^-1^, *p* = 0.010) in the left putamen compared to the H-Y 1-2 subgroup. Notably, C_deoxy_ levels were significantly elevated in various subregions of the left putamen (including the anterior, posterior, and lateral dorsal areas) in the H-Y 2.5-3 subgroup (*p* < 0.05) (Table **2**).

### 3.3 Correlation between Cerebral Hemodynamic Parameters and Disease Progression

In the entire group of patients with PD, no significant correlation was found between oxygen metabolism parameters in various brain regions (C_deoxy_, OEF_rel_, and dCBV) and the UPDRS scores (*p* > 0.05). Several positive correlations were observed in the H-Y stage (1-2) group. Specifically, C_deoxy_ (r = 0.39, *p* < 0.05, r = 0.42, *p* < 0.05), OEF_rel_ (r = 0.49, *p* < 0.01), and R2* values in the substantia nigra (r = 0.52, *p* < 0.01) and red nucleus (r = 0.48, *p* < 0.01) were all positively correlated with the UPDRS scores. Conversely, in the H-Y stage (2.5-3) group, significant correlations were observed mainly in the striatal structures. These included C_deoxy_ (r = 0.41, *p* < 0.05) and dCBV (r = 0.43, *p* < 0.05) in the left dorsolateral putamen, and OEF_rel_ (r = 0.41, *p* < 0.05) in the posterior right caudate, all of which were positively correlated with the UPDRS scores (Fig. **4**).

### 3.4 Correlation Between Cerebral Hemodynamic Parameters and Dopamine Transporter Availability as well as Glucose Metabolism

C_deoxy_ and dCBV levels are not significantly associated with dopamine transporter availability or glucose metabolism. However, OEF_rel_ exhibited a positive correlation with glucose metabolism in the left putamen (r = 0.64, *p* < 0.01) and right caudate (r = 0.56, *p* < 0.05). Additionally, the R2* values demonstrated a positive correlation with dopamine transporter availability in the left caudate (r = 0.45, *p* < 0.05) and with glucose metabolism in multiple regions, including the left caudate (r = 0.49, *p* < 0.05), left pre-caudate (r = 0.46, *p* < 0.05), and left post-caudate (r = 0.59, *p* < 0.01) (Fig. **5**).

## 4 DISCUSSION

This study employed qMRI to investigate hemodynamics and blood oxygen metabolism in key brain regions in patients with PD, with a particular focus on the nigrostriatal system. These findings revealed that the oxygen metabolism patterns were similar between patients with PD and HCs. Specifically, the C_deoxy_ and OEF_rel_ levels were lower in the caudate nucleus than in the putamen, indicating a higher metabolic profile in the putamen. Importantly, patients with PD exhibit lower levels of C_deoxy_ and dCBV in the bilateral caudate nucleus and frontal cortex than controls, suggesting compensatory increases in blood flow and metabolic activity in the striatal-frontal pathway. Conversely, elevated C_deoxy_ and OEF_rel_ levels in the substantia nigra indicated significant tissue hypoxia in this region. Moreover, the left putamen showed more C_deoxy_ in the mid-to-late stages of the disease than in the early stages, and this area was linked to motor symptoms. This result suggests that the left putamen may be the first brain region to exhibit signs of decompensated hypoxia during disease progression, thereby highlighting its importance in tracking such progression.

Physiological variables such as OEF, cerebral blood flow (CBF), and cerebral metabolic rate of oxygen (CMRO2) are widely used to assess the oxygen utilization, blood supply, and overall energy consumption status of the brain [40-43]. Advancements in MRI technology have enabled the development of non-invasive quantitative methods based on paramagnetism to predict OEF, thereby providing a substitute for invasive PET [28, 44]. In AD, qMRI studies have shown that age-corrected regional OEF_rel_ remains stable, whereas CMRO2 significantly decreases in specific cortical areas [45-47]. Similarly, research using the MEGRE technique to model blood oxygen metabolism parameters (OEF_rel_, dCBV, and C_deoxy_) in normal aging populations has demonstrated that these parameters do not decline with age [26]. In our study, both patients with PD and healthy controls exhibited lower cerebral hemodynamic parameters, particularly C_deoxy_, in the caudate nucleus than in the putamen, with a gradient of decreasing oxygen metabolism from the caudate to the putamen, consistent with findings from dopamine metabolic pathway studies [48]. Especially in the substantia nigra, there was a positive correlation with the degree of early motor symptoms, patients with PD showed a notable increase in the relative OEF_rel_.

In contrast, no significant OEF_rel_ changes are observed in other extrapyramidal-associated regions, such as the putamen, caudate nucleus, or frontal cortex. The substantia nigra underwent early dopaminergic neuron degeneration [49-51]. Surviving neurons may compensate for hypermetabolism, increase oxygen demand and elevate OEF_rel_. Concurrently, reduced nigral blood flow, potentially due to vascular dysregulation or capillary loss [29, 39], may exacerbate the oxygen supply-demand mismatch, driving OEF_rel_ elevation independently of changes in the cerebral metabolic rate of CMRO2 [29].

Notably, the C_deoxy_ values demonstrated greater variability in the core brain regions of patients with PD. Compared with the control group, the PD group demonstrated elevated C_deoxy_ levels in the substantia nigra. In contrast, bilateral caudate nuclei (particularly in the anterior subregions) and frontal cortical regions exhibited significantly reduced C_deoxy_ and dCBV values. In this study, patients with PD exhibited significantly more prominent alterations in C_deoxy_ values than in R2* values when compared with age-matched healthy controls. Crucially, R2* serves as an established neuroimaging biomarker [52-54] associated with iron deposition in Parkinsonian pathophysiology, primarily reflecting static tissue iron content [45, 55]. This finding is particularly compelling, as C_deoxy_ levels in brain tissue are closely linked to cerebral oxygen consumption [27, 56]. During neuronal activation, oxyhemoglobin levels rise rapidly, whereas C_deoxy_ levels gradually decline [29, 57]. An increase in C_deoxy_ suggests tissue hypoxia, potentially due to insufficient blood flow, reduced oxygen supply, or heightened local brain metabolism and functional activity [29, 58]. Moreover, in certain brain function-related disorders, elevated C_deoxy_ levels may directly reflect pathological changes, indicating tissue dysfunction or damage [59].

Our study revealed significantly elevated C_deoxy_ levels in the substantia nigra of patients with PD, with these elevations correlating with motor symptom severity during the early stages of the disease. This finding suggests that the nigral tissue undergoes increased oxygen utilization and early hypoxia during the progression of PD. Previous studies have extensively implicated hypoxia-related mechanisms in PD, including mitochondrial dysfunction [60, 61] and selective degeneration of dopaminergic neurons in the substantia nigra pars compacta (SNc) triggered by ɑ-synuclein overexpression [62, 63]. These pathological processes align with the observed alterations in OEF, further supporting the hypothesis that hypoxia in the substantia nigra during earlystage PD may exacerbate neurodegenerative pathology [64, 65]. Other studies have suggested that the dopaminergic neurons in the SNc are sensitive to hypoxic conditions [64, 66].

In contrast, there was a significant decrease in C_deoxy_ levels in the bilateral caudate nucleus, particularly in the anterior portion and frontal cortex, in the PD group [18]. This reduction in C_deoxy_ levels is often indicative of enhanced activity in specific brain regions, which is consistent with previous findings from FDG-PET metabolic network studies of PD [67, 68]. Numerous studies have validated that the characteristic features of the PD-related pattern (PDRP) include increased glucose metabolism in the striatum, thalamus, pons, and cerebellum [24, 69]. The reasons for increased metabolism in the putamen and caudate nucleus of patients with PD remain unclear. Studies have suggested that these findings may stem from factors, such as a compensatory response to dopaminergic neuron loss [38] and an imbalance between excitatory (glutamate) and inhibitory (GABA) neurotransmitters [70].

Additionally, oxidative stress, neuroinflammation, and mitochondrial dysfunction may prompt increased oxygenation in neurons and glial cells to meet energy demands [18, 71]. Furthermore, oxygen metabolism in the left putamen was closely correlated with clinical motor symptoms in the later stages of the disease, whereas in the early stages, this correlation was more pronounced in the red nucleus and substantia nigra. Several previous studies have confirmed that functional deficits in the putamen are associated with motor symptoms of PD [72]. Johansson *et al*. reported a negative correlation between putamen volume and disease duration [73]. Additionally, functional MRI studies have shown that left putamen dysfunction is correlated with motor symptoms in PD [74].

To further clarify the relationship between the oxygen metabolism patterns in PD and PDRP, we conducted a multimodal PET data correlation analysis. Although other oxygenation parameters in the brain tissue did not show significant correlations with dopamine transport or glucose metabolism, this may be related to the sample size and stage bias of the included patients. Nonetheless, in the PD group, OEF_rel_ in the left putamen and right caudate nucleus was positively correlated with dopamine transport and glucose metabolism in these brain regions, suggesting a possible association between oxygen metabolism and the pathological mechanisms of the disease. Additionally, in this study, we utilized qMRI to extract R2* values, which represent iron deposition [53, 75]. Previous studies have reported elevated R2* values in the substantia nigra of patients [34, 76]. Our results indicated higher R2* values in the substantia nigra compared to controls, which positively correlated with UPDRS scores, dopamine transport, and glucose metabolism in the putamen and caudate nucleus.

The limitations of this study include the focus on subregion segmentation of the striatum without a detailed analysis of the substantia nigra. The absence of Arterial Spin Labeling (ASL) data also precludes the extraction of CBF and CMRO2, so restricting the comprehensiveness of our brain oxygen metabolism profile. Future research should address these gaps by increasing the sample size and also by performing subtype analyses of PD, including tremor and non-tremor variants. Furthermore, we intend to utilize machine learning techniques to integrate multimodal imaging data, create a comprehensive metabolic map of PD, and examine the metabolic profile differences across subtypes and disease stages.

## CONCLUSION

This study highlights the emerging role of qMRI in detecting hemodynamic and metabolic disturbances in PD, particularly its sensitivity to nigrostriatal system dysfunction. Elevated levels of C_deoxy_, OEF_rel_, and R2* observed in the substantia nigra, a hallmark pathological region in PD, indicate localized metabolic stress characterized by increased oxygen extraction, tissue hypoxia, and iron deposition. Reduced C_deoxy_ and dCBV levels in the caudate nucleus and frontal cortex suggested impaired oxygen delivery or disrupted neurovascular coupling in these interconnected regions, highlighting the systemic nature of metabolic dysregulation in patients with PD. By providing a non-invasive window for early-stage pathophysiology and therapeutic efficacy evaluation, the brain oxygen metabolism parameters obtained from qMRI show great promise as complementary imaging biomarkers for diagnosing PD and tracking its progression.

## Figures and Tables

**Fig. (1):**
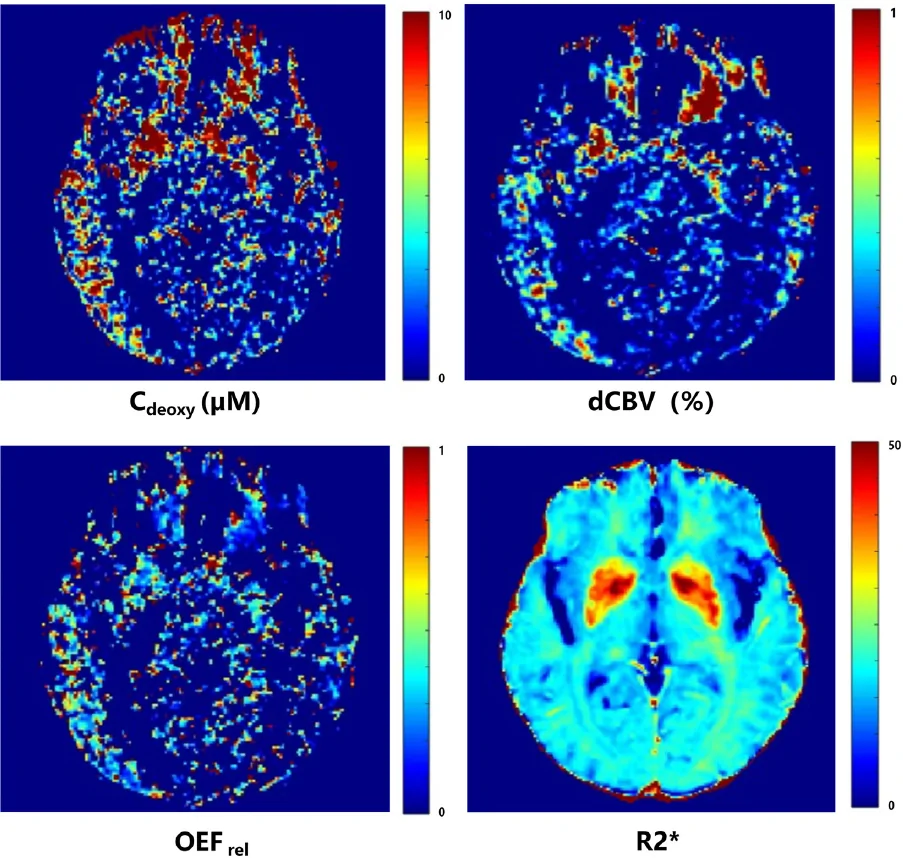
C_deoxy_, dCBV, OEF_rel_, and R2*maps from a PD patient. dCBV, deoxygenated cerebral blood volume; C_deoxy_, deoxyhemoglobin concentration; OEF, oxygen extraction fraction.

**Fig. (2):**
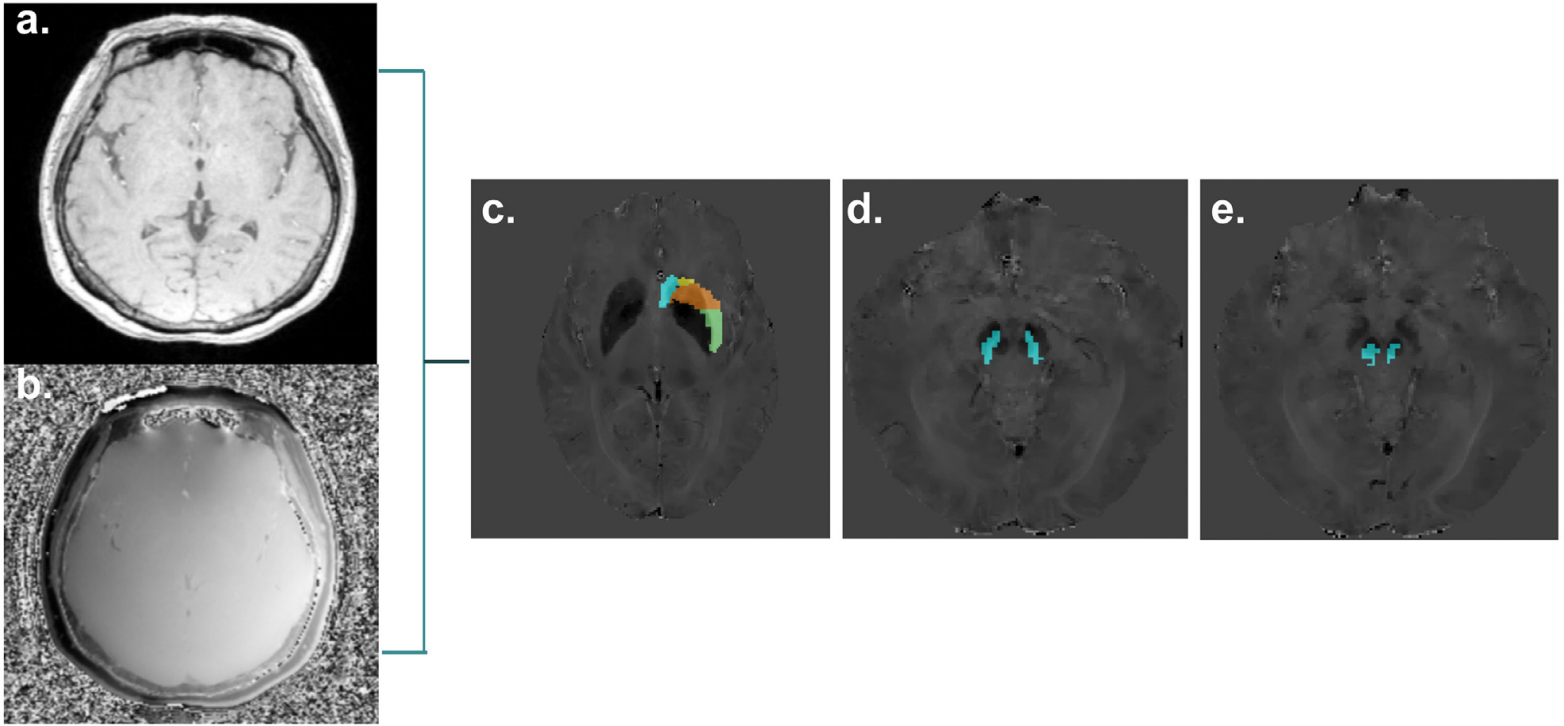
The segmentation maps of the striatum and substantia nigra. Phase maps extracted from the GRE sequence were aligned with the T1 images and registered to a standardized brain template for validation (**a**, **b**). Custom MATLAB code was used to extract the OEF, dCBV, C_deoxy_, and R2* values from the standardized ROIs, with the corresponding ROIs selected from the automatically segmented basal ganglia images (**c**)—2D regions of the striatum. The left pre-commissural striatum comprises the left ventral putamen (orange), left dorsal putamen (yellow), and caudate (blue). The left postcommissural striatum includes the putamen (green) and the caudate. The substantia nigra and red nuclei were separately segmented (**d**, **e**).

**Fig. (3):**
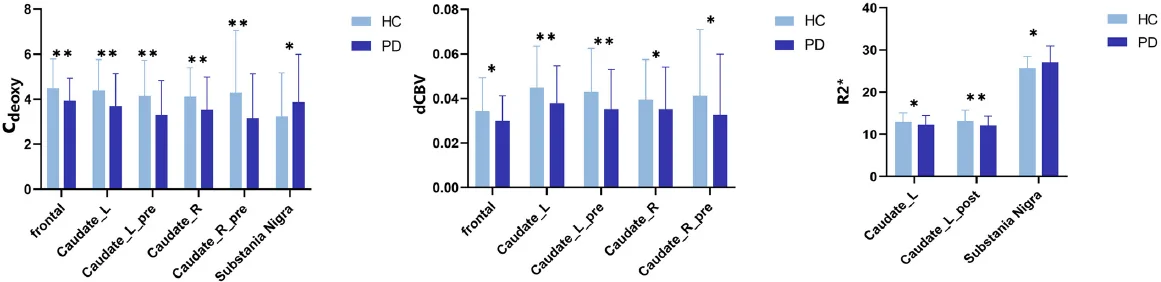
Differences in C_deoxy_, OEF_rel_, dCBV, and R2* values in brain regions between PD and HC groups. *p < 0.05, **p < 0.01. **Abbreviations**: PD, Parkinson's disease; HC, healthy control; dCBV, deoxygenated cerebral blood volume; C_deoxy_, deoxyhemoglobin concentration.

**Fig. (4):**
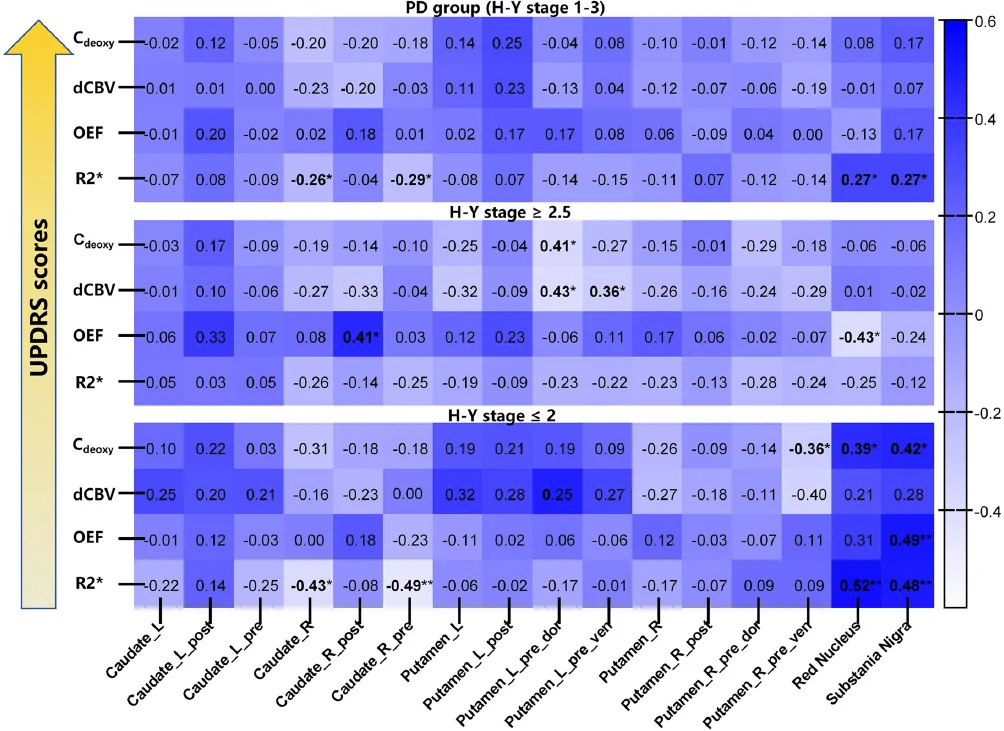
The correlation between cerebral oxygen metabolism in key brain regions and disease progression. The image displays three correlation matrices illustrating the associations between the metabolic parameters of various brain regions (C_deoxy_, dCBV, OEF, and R2*) and the total UPDRS score across the three subgroups stratified by the H-Y stage. Each cell within the matrix represents the correlation coefficient between a given pair of variables: *p < 0.05, **p < 0.01. **Abbreviations**: OEF, oxygen extraction fraction; dCBV, deoxygenated cerebral blood volume; C_deoxy_, deoxyhemoglobin concentration; H-Y, Hoehn-Yahr; UPDRS, Unified Parkinson's Disease Rating Scale.

**Fig. (5):**
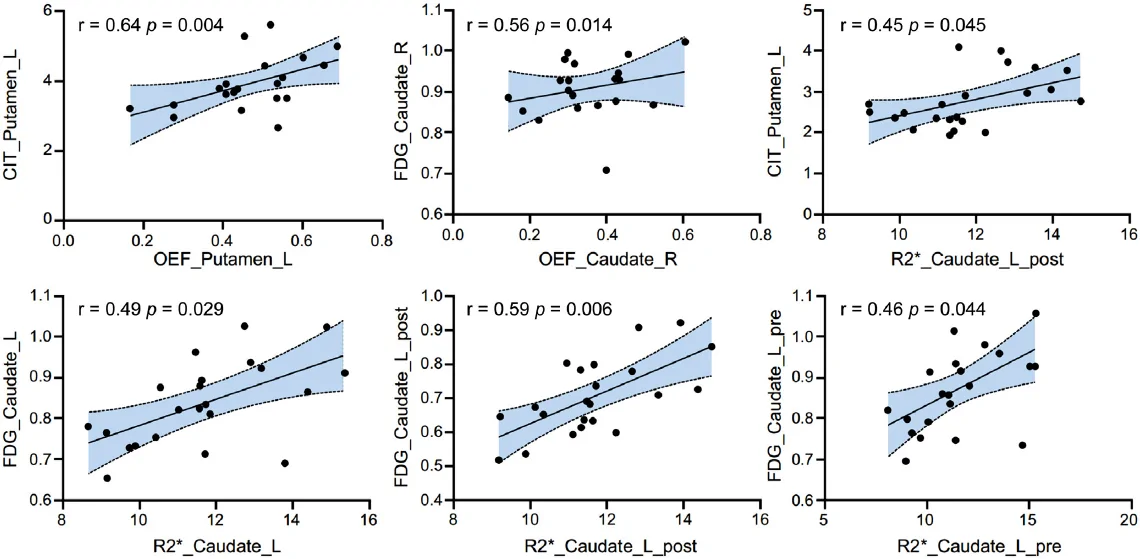
The correlation between cerebral oxygen metabolism, dopaminergic transport, and glucose uptake. The figure is plotted based on the Pearson correlation at the ROI level between the qMRI values (OEF_rel_, R2*) and [18F] FDG PET, as well as DAT-PET. OEF_rel_ values were positively correlated with DAT SUVR exclusively in the left putamen, whereas a positive correlation with FDG SUVR was noted only in the right caudate. Additionally, R2* values showed a positive correlation with DAT SUVR in the left putamen and a positive correlation with FDG SUVR in the left caudate and left pre/post-commissural regions of the caudate (*p* < 0.05), indicating statistical significance.

**Table 1:** Demographic and clinical characteristics of PD patients and normal control participants.

**Characteristic**	**PD**	**HC**	***p* Value**
Number	73	67	NA
Year (mean ± SD)	61.10 ± 8.58	58.99 ± 8.74	0.16
Sex (female, %)	41 (56.2%)	32 (47.8%)	0.32
H&Y (M)	2(1.5,2.5)	NA	NA
UPDRS III	38.48 ± 16.09	NA	NA
DAT+FDG PET (n)	22	NA	NA

**Abbreviations**: PD, Parkinson's disease; HC, healthy control; H-Y, Hoehn-Yahr; UPDRS, Unified Parkinson's Disease Rating Scale.

**Table 2:** Differences in Cdeoxy, OEFrel, dCBV, and R2* values in brain regions between PD disease progression subgroups.

**Variable PD Group (HY 1-2 *vs.* HY 2.5-3)**	**Region (Median)**	***p***
Cdeoxy (µM) Contrast: H-Y 1-2 group < H-Y 2.5-3 group	Putamen_L (3.77 *vs.* 5.33)	0.001**
	Putamen_L_post (3.03 *vs.* 4.97)	0.012*
	Putamen_L_pre_dor (5.63 *vs.* 4.97)	0.022*
	Putamen_L_pre_ven (4.02 *vs.* 5.97)	0.001**
dCBV Contrast: H-Y 1-2 group < H-Y 2.5-3 group	Putamen_L_pre_ven (0.036 *vs.* 0.046)	0.028*
OEF _rel_ Contrast: H-Y 1-2 group < H-Y 2.5-3 group	Putamen_L_pre_dor (0.32 *vs.* 0.42)	0.037*
R2*(S^-1^) Contrast: H-Y 1-2 group < H-Y 2.5-3 group	Putamen_L (22.31 *vs.* 23.63)	0.010*
	Putamen_L_post (21.73 *vs.* 22.77)	0.009**
	Putamen_L_pre_ven (23.21 *vs.* 24.27)	0.024*
	Putamen_R_post (20.40 *vs.* 21.65)	0.034*
	Red Nucleus (23.37 *vs.* 24.52)	0.026*
	Substania Nigra (26.04 *vs.* 27.75)	0.031*

**Note**: *p < 0.05, **p < 0.01. The description of the statistical results follows the format of median and interquartile range, with the median presented first, followed by parentheses.

**Abbreviations**: PD, Parkinson's disease; *vs*., *versus*; H-Y, Hoehn-Yahr; dor, dorsal; ven, ventral; OEF, oxygen extraction fraction; dCBV, deoxygenated cerebral blood volume; C_deoxy_, deoxyhaemoglobin concentration

## Data Availability

The data that support the findings of this study are available from the corresponding author, (PH and KW), upon reasonable request.

## LIST OF ABBREVIATIONS

CBF: Cerebral Blood Flow
FOV: Field Of View
MNI: Montreal Neurological Institute
MRI: Magnetic Resonance Imaging
PET: Positron Emission Tomography
PVC: Partial Volume Correction
ROIs: Regions Of Interest
SUVR: Standardized Uptake Value Ratio
TR: Repetition Time

## STANDARDS OF REPORTING

STROBE guidelines were followed.
