# Supplementary material for: Evaluation of Hemodynamic and Blood Oxygen Metabolism Alterations in Parkinson's Disease Using Quantitative MRI
Source: Curr Neuropharmacol. 2025 Dec 2;24(11):1639–49. doi: 10.2174/011570159X385539250706094824 (PMC13647658; doi:10.2174/011570159X385539250706094824)
Supplement: Supplementary file 1 [file CN-24-11-1639_SD1.pdf]

Supplementary Material

Evaluation of Hemodynamic and Blood Oxygen Metabolism Alterations in Parkinson's Disease Using Quantitative MRI

Xinyi Lv[1,2], Rong Ye[1], Jie Wen[3], Qiang Xie[4], Yuhua Chen[2], Panpan Hu[1,*] and Kai Wang[1,*]

[1]Department of Neurology, The First Affiliated Hospital of Anhui Medical University, The School of Mental Health and Psychological Sciences, Anhui Medical University, Hefei, 230032, China; [2]Department of Neurology, The First Affiliated Hospital of USTC, Division of Life Sciences and Medicine, University of Science and Technology of China, Hefei, Anhui 230001, China; [3]Department of Radiology, The First Affiliated Hospital of USTC, Division of Life Sciences and Medicine, University of Science and Technology of China, Hefei, Anhui 230001, China; [4]Department of Nuclear Medicine, The First Affiliated Hospital of USTC, Division of Life Sciences and Medicine, University of Science and Technology of China, Hefei, Anhui 230001, China

Table A1. Differences in C_{deoxy}, OEF_{rel}, dCBV, and R2* values in Brain regions between PD and HC groups.

	Cdeoxy (μM)			dCBV（%）			OEF_{rel}			R2*(S^{-1})		
	PD	HC	*p*	PD	HC	*p*	PD	HC	*p*	PD	HC	*p*
Caudate_L	3.5(2.49,4.68)	4.39(3.22,5.4)	0.001**	0.03(0.02,0.05)	0.04(0.03,0.06)	0.004**	0.35(0.23,0.48)	0.35(0.27,0.46)	0.775	12.11(10.55,13.77)	13.23(11.26,14.99)	0.016*
Caudate_L_post	4.31(3.06,5.55)	4.98(3.37,7.19)	0.085	0.04(0.03,0.05)	0.05(0.04,0.06)	0.146	0.33(0.25,0.48)	0.36(0.26,0.53)	0.334	11.72(10.32,13.45)	12.41(11.37,15.22)	0.004**
Caudate_L_pre	3.12(2.09,4.36)	3.9(2.85,5.53)	0.002**	0.03(0.02,0.05)	0.04(0.03,0.07)	0.003**	0.32(0.2,0.47)	0.34(0.21,0.43)	0.941	12.22(10.14,13.57)	12.83(11.33,14.8)	0.053
Caudate_R	3.04(2.72,4.47)	3.93(3.25,5.27)	0.006**	0.03(0.02,0.04)	0.04(0.03,0.05)	0.026*	0.32(0.25,0.42)	0.39(0.29,0.48)	0.091	14.75(12.67,16.09)	15.44(13.97,16.72)	0.066
Caudate_R_post	4.93(3.49,7.27)	4.42(3.22,7.02)	0.501	0.04(0.02,0.06)	0.04(0.02,0.06)	0.642	0.37(0.29,0.51)	0.38(0.25,0.57)	0.866	16.28(14.15,18.02)	16.77(14.73,19.3)	0.117
Caudate_R_pre	2.83(1.75,4.17)	3.91(2.39,5.45)	0.004**	0.03(0.01,0.04)	0.03(0.02,0.06)	0.034*	0.24(0.16,0.39)	0.32(0.2,0.48)	0.067	14.34(12.69,16.76)	14.81(13.57,17.53)	0.066
Putamen_L	4.6(3.54,6.29)	4.5(3.35,6.62)	0.778	0.04(0.03,0.06)	0.04(0.03,0.06)	0.477	0.44(0.36,0.53)	0.41(0.29,0.54)	0.613	23.02(21.59,24.78)	23.28(21.7,24.89)	0.490
Putamen_L_post	4.04(2.68,5.79)	3.74(2.63,5.45)	0.693	0.04(0.02,0.05)	0.04(0.02,0.06)	0.721	0.42(0.31,0.53)	0.35(0.27,0.52)	0.272	22.36(20.85,24.08)	22.9(21.34,24.55)	0.221
Putamen_L_pre_dor	5.82(4.38,8.11)	5.59(3.87,8.03)	0.823	0.05(0.04,0.08)	0.05(0.04,0.08)	0.519	0.36(0.25,0.5)	0.34(0.19,0.56)	0.389	24.4(22.19,25.73)	24(22.38,26.51)	0.798
Putamen_L_pre_ven	4.5(3.41,6.4)	4.83(3.7,6.26)	0.547	0.04(0.03,0.06)	0.05(0.03,0.07)	0.398	0.38(0.29,0.53)	0.37(0.29,0.56)	0.741	23.72(22.47,25.65)	24.08(21.69,25.44)	0.624
Putamen_R	4.3(3.38,5.43)	4.64(3.26,6.63)	0.506	0.04(0.03,0.05)	0.04(0.03,0.06)	0.126	0.41(0.34,0.57)	0.43(0.31,0.54)	0.633	22.44(20.69,23.87)	22.35(20.81,23.9)	0.886
Putamen_R_post	3.84(2.66,5.31)	3.51(2.21,5.47)	0.536	0.03(0.02,0.05)	0.03(0.02,0.05)	0.998	0.39(0.29,0.56)	0.4(0.24,0.52)	0.546	21.13(19.74,23)	20.94(19.79,22.57)	0.525
Putamen_R_pre_dor	5.16(3.77,6.25)	5.76(4.33,7.02)	0.129	0.04(0.03,0.07)	0.05(0.04,0.08)	0.092	0.36(0.21,0.51)	0.34(0.27,0.49)	0.715	23.32(21.5,25.07)	23.22(21.46,25.05)	0.627
Putamen_R_pre_ven	3.65(2.78,5.12)	4.49(2.59,6.61)	0.177	0.03(0.02,0.04)	0.04(0.03,0.07)	0.047*	0.41(0.31,0.56)	0.42(0.3,0.6)	0.989	22.94(20.89,24.1)	23.03(21.86,25.43)	0.183
Red Nucleus	3.05(1.5,4.66)	2.73(1.35,4.09)	0.274	0.03(0.01,0.04)	0.02(0.01,0.04)	0.169	0.3(0.16,0.5)	0.26(0.16,0.44)	0.342	23.59(22.6,25.92)	23.64(22.21,25.9)	0.856
Substania Nigra	3.40(2.45,5.94)	2.98(1.67,4.71)	0.038*	0.03(0.02,0.05)	0.03(0.01,0.05)	0.063	0.31(0.18,0.53)	0.24(0.14,0.39)	0.047*	26.67(24.47,29.04)	25.4(23.23,27.44)	0.016*
Thalamus	3.59(2.76,4.44)	3.29 (2.68,4.17)	0.347	0.03(0.02,0.04)	0.03(0.02,0.04)	0.296	0.45(0.34,0.56)	0.45(0.34,0.58)	0.828	18.07(17.36,19.02)	18.52(17.30,19.69)	0.343
Cerebellum	3.78 (2.56,4.75)	3.32 (2.32,4.53)	0.561	0.03 (0.02,0.04)	0.03 (0.02,0.04)	0.357	0.40 (0.32.0.51)	0.39 (0.30,0.50)	0.567	15.05 (13.57,15.78)	14.50 (13.21, 15.02)	0.654
frontal	3.77(3.32,4,23)	4.20(3.66,4.82)	0.003**	0.03(0.02,0.03)	0.03(0.03,0.04)	0.045*	0.57(0.48,0.70)	0.56(0.47,0.65)	0.467	16.71(16.10,17.50)	17.20(16.55,17.85)	0.066
occipital	3.71(3.28,4.08)	3.61(3.20,4.49)	0.728	0.03(0.02,0.03)	0.03(0.03,0.04)	0.660	0.54(0.44,0.61)	0.53(0.45,0.65)	0.884	20.55(19.97,21,43)	20.23(19.43,20.93)	0.062
HIPP	4.76(3.88,5.52)	4.49(3.44,5.62)	0.823	0.04(0.04,0.05)	0.04(0.03,0.06)	0.832	0.40(0.33,0.51)	0.41(0.30,0.51)	0.932	18.10(16.71,19.04)	18.18(17.05,19.06)	0.621

*$P < 0.05$, **$P < 0.01$. The description of the statistical results follows the form of median and interquartile range, with the median before the parentheses, the first number in the parentheses being the 25% quartile, and the second number the 75% quartile.

Abbreviations : PD: Parkinson's disease; HC: healthy control; dor: dorsal; ven: ventral; OEF: oxygen extraction fraction; dCBV: deoxygenated cerebral blood volume; Cdeoxy : deoxyhemoglobin concentration.

1875-6190/26 $65.00+.00 © 2026 Bentham Science Publishers
